# Supplementary material for: Differences in Free-Living Patterns of Sedentary Behaviour between Office Employees with Diabetes and Office Employees without Diabetes: A Principal Component Analysis for Clinical Practice
Source: Int J Environ Res Public Health. 2022 Sep 27;19(19):12245. doi: 10.3390/ijerph191912245 (PMC9566776; doi:10.3390/ijerph191912245)
Supplement: Supplementary file 1 [file ijerph-19-12245-s001.zip › ijerph-1860295-supplementary.pdf]

Table S1. Abbreviations of the variables measured in the study.

Abbreviation	Variable
Diabetic	Adults diagnosed with T2D according to International Criteria
Age	Patient’s age (numeric)
Sex	Patient’s sex (categorical: M—male and F—female).
BMI	Patient’s body mass index (numeric: <18.5 kg/m2 underweight, from 18.5 to 25 kg/m2 normal, 25 to 30 kg/m2 overweight, and >30 kg/m2 obese).
**W**	**Weekday – Total time**
W-LIPA	Weekday light-intensity physical activity (minutes/day)
W-MVPA	Weekday moderate to vigorous physical activity (minutes/day)
W-Seden	Weekday sedentary time (minutes/day)
W-Stand	Weekday standing time (minutes/day)
W-B20	Weekday sedentary breaks <20 min (number/day)
W-B2040	Weekday sedentary breaks of 20-40 min (number/day)
W-B4060	Weekday sedentary breaks of 40-60 min (number/day)
W-B60	Weekday sedentary breaks >60 min (number/day)
W-T20	Weekday time spent in sedentary bouts <20 min (minutes/day)
W-T2040	Weekday time spent in sedentary bouts 20-40 min (minutes/day)
W-T4060	Weekday time spent in sedentary bouts 40-60 min (minutes/day)
W-T60	Weekday time spent in sedentary bouts >60 min (minutes/day)
W-TB	Weekday total sedentary breaks (number/day)
W-TT	Weekday total sedentary time (minutes/day) weekday
**WW**	**Weekday - Working time**
WW-LIPA	Light-intensity physical activity during working hours (minutes/day)
WW-MVPA	Moderate to vigorous physical activity during working hours (minutes/day)
WW-Seden	Sedentary time during working hours (minutes/day)
WW-Stand	Standing time during working hours (minutes/day)
WW-B20	Sedentary breaks <20 min during working hours (number/day)
WW-B2040	Sedentary breaks of 20-40 min during working hours (number/day)
WW-B4060	Sedentary breaks of 40-60 min during working hours (number/day)

WW-B60	Sedentary breaks >60 min during working hours (number/day)
WW-T20	Time spent in sedentary bouts <20 min during working hours (minutes/day)
WW-T2040	Time spent in sedentary bouts of 20-40 min during working hours (minutes/day)
WW-T4060	Time spent in sedentary bouts of 40-60 min during working hours (minutes/day)
WW-T60	Time spent in sedentary bouts >60 min during working hours (minutes/day)
WW-TB	Total sedentary breaks (number/day)
**WNW**	**Weekday – Non-Working time**
WNW-LIPA	Light-intensity physical activity during non-working hours (minutes/day)
WNW-MVPA	Moderate to vigorous physical activity during non-working hours (minutes/day)
WNW-Seden	Sedentary time during non-working hours (minutes/day)
WNW-Stand	Standing time during non-working hours (minutes/day)
WNW-B20	Sedentary breaks <20 min during non-working hours (number/day)
WNW-B2040	Sedentary breaks of 20-40 min during non-working hours (number/day)
WNW-B4060	Sedentary breaks of 40-60 min during non-working hours (number/day)
WNW-B60	Sedentary breaks >60 min during non-working hours (number/day)
WNW-T20	Time spent in sedentary bouts <20 min during non-working hours (minutes/day)
WNW-T2040	Time spent in sedentary bouts of 20-40 min during non-working hours (minutes/day)
WNW-T4060	Time spent in sedentary bouts of 40-60 min during non-working hours (minutes/day)
WNW-T60	Time spent in sedentary bouts >60 min during non-working hours (minutes/day)
WNW-TB	Total sedentary breaks during non-working hours (minutes/day)
**WD**	**Weekend**
WD-LIPA	Weekend light-intensity physical activity (minutes/day)
WD-MVPA	Weekend moderate to vigorous physical activity (minutes/day)
WD-Seden	Weekend sedentary time (minutes/day)
WD-Stand	Weekend standing time (minutes/day)
WD-B20	Weekend sedentary breaks <20 min (number/day)
WD-B2040	Weekend sedentary breaks of 20-40 min (number/day)
WD-B4060	Weekend sedentary breaks of 40-60 min (number/day)

WD-B60	Weekend sedentary breaks >60 min (number/day)
WD-T20	Weekend time spent in sedentary bouts <20 min (minutes/day)
WD-T2040	Weekend time spent in sedentary bouts of 20-40 min (minutes/day)
WD-T4060	Weekend time spent in sedentary bouts of 40-60 min (minutes/day)
WD-T60	Weekend time spent in sedentary bouts >60 min (minutes/day)
WD-TB	Weekend total sedentary breaks (number/day)

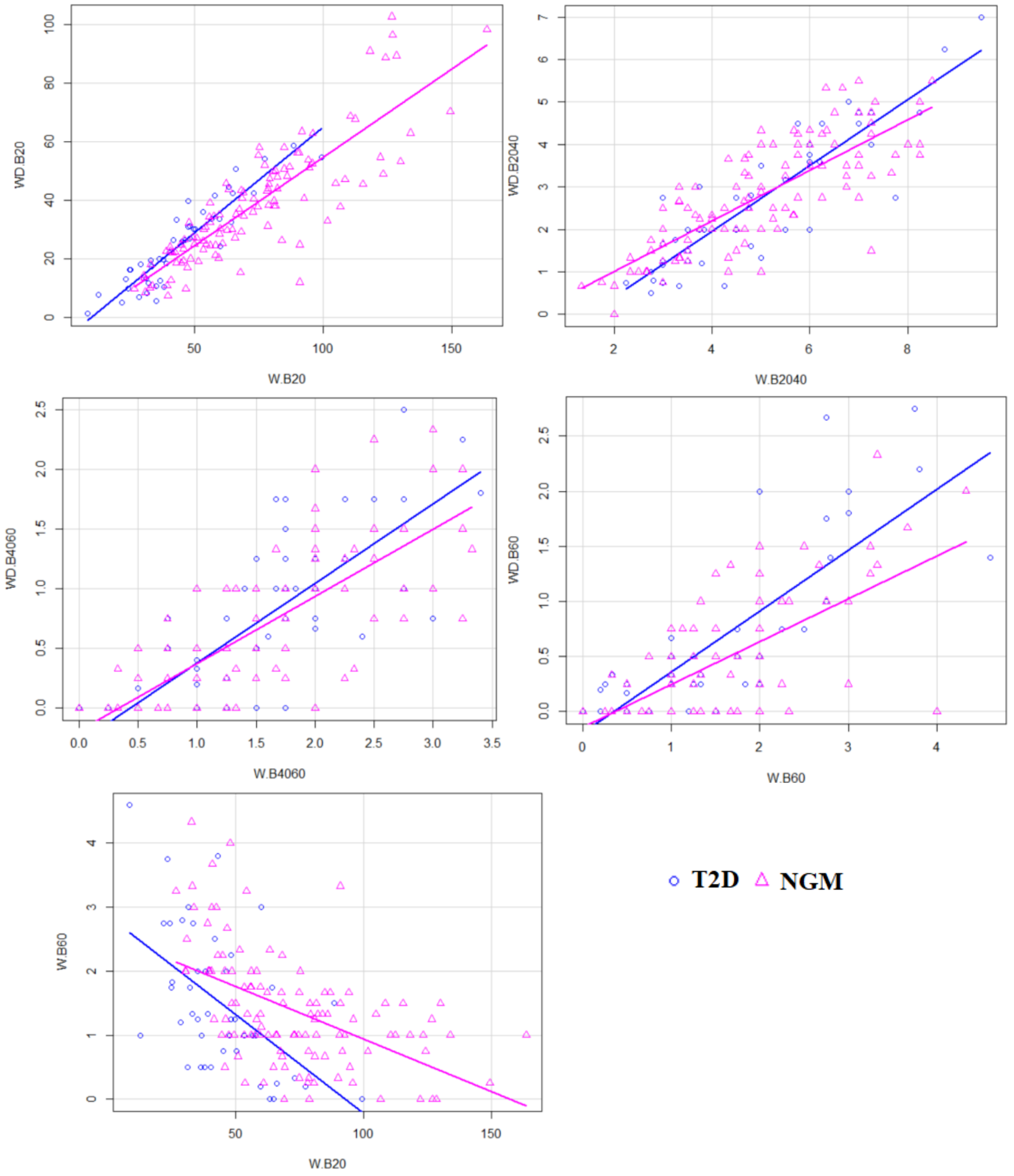

Figure S1. Scatter plots between the variables of the SB pattern on weekdays and at weekends.
